# Supplementary material for: Comparative multiomics analysis of cell physiological state after culture in a basket bioreactor
Source: Sci Rep. 2022 Nov 23;12:20161. doi: 10.1038/s41598-022-24687-4 (PMC9686226; doi:10.1038/s41598-022-24687-4)

3666068.1:28339882:28340169:+@NW_023666068.1:28340509:28341349:+@NW_023666068.1:28341236:2834

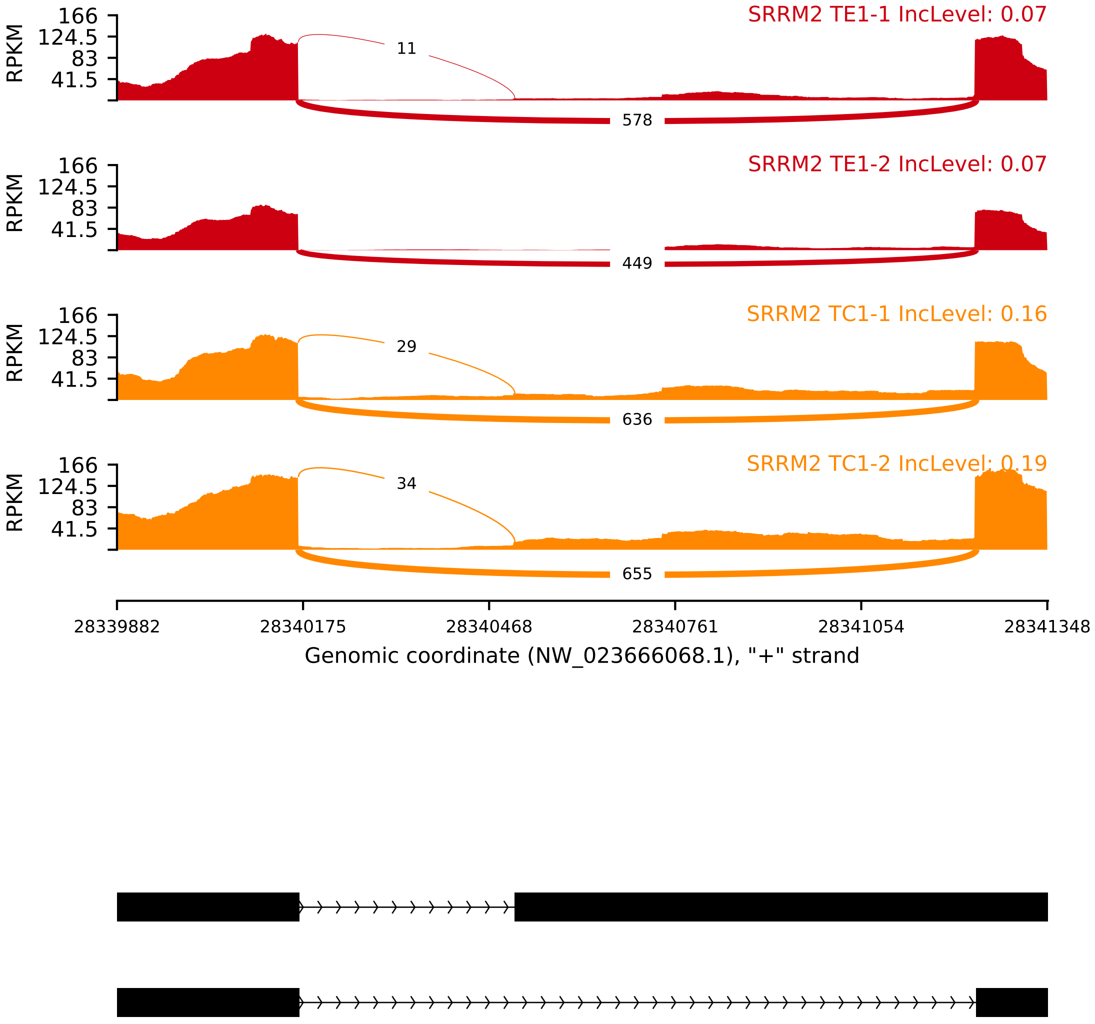

Supplement: Supplementary file 1 — Supplementary Information 1. [file 41598_2022_24687_MOESM1_ESM.zip › raw data/Transcriptomic raw data/7.AS/2.dasplot/TE1vsTC1/A3SS/20_SRRM2_NW_023666068.1_28339882_28340169_+@NW_023666068.1_28340509_28341349_+@NW_023666068.1_28341236_28341349_+.pdf]
